# Gender Related Differences in Response to “In Favor of Myself” Wellness Program to Enhance Positive Self & Body Image among Adolescents

**DOI:** 10.1371/journal.pone.0091778

**Published:** 2014-03-11

**Authors:** Moria Golan, Noa Hagay, Snait Tamir

**Affiliations:** 1 Shahaf, Community Services for the Management of Weight-Related Problems, Tel Aviv, Israel; 2 School of Nutritional Sciences, Tel Hai College, Upper Galilee, Israel; 3 School of Nutritional Sciences, The Hebrew University of Jerusalem, Rehovot, Israel; Scientific Directorate, Bambino Hospital, Italy

## Abstract

**Background:**

Physical, neurological and psychological changes are often experienced differently by male and female adolescents. Positive self-esteem, emotional well-being, school achievements, and family connectedness are considered as protective factors against health-compromising behaviors. This study examines the gender differences in respect to the effect of a school-based interactive wellness program – “In Favor of Myself” – on self-image, body image, eating attitudes and behaviors of young adolescents.

**Methods:**

Two hundred and ten adolescents (mean age 13.5) participated in the intervention group, 55% were girls and 45% boys. Program consisted of eight 90-minutes structured sessions integrated into a regular school coping skills curriculum. The program focused on self-esteem, self-image, body image, media literacy and cognitive dissonance. The overall impact of the program and the study protocol were previously published.

**Results:**

Overall, there are gender related differences in respect to body image and self-image in young adolescents in response to “In Favor of Myself”. Compared to boys, girls reported at baseline higher self-esteem, being more contingent by appearance, and their self-image was more influenced by popularity, appearance, interpersonal communication and admired people. Furthermore girls presented greater gap between current body figure and perceived ideal figure. Not only were girls more dissatisfied with their body, but they were more active in attempts to become and/or remain “thin”. At program termination, gender × time effect was detected in reduction of self-worth contingent by others, change in importance given to achievements at schools, parents' perceptions, as well as the impact of comparisons to friends and family members on self-image.

**Conclusions:**

Girls exhibited more gains than boys from ‘In Favor of Myself’ which raise the questions about how effective would be the program when delivered in mixed gender groups vs. mono-gender groups.

## Introduction

School-based intervention is a viable mechanism for widespread intervention which has the particular advantage of near universal enrollment of children and adolescents. School-based programs can facilitate change in the environment as well as encourage adopting a healthy lifestyle [1].

Previous findings on eating disorder prevention programs have been mixed. Paxton [2] reported that about half the school-based curricula have been shown to have positive impact on some aspects of body image. A smaller proportion has produced changes in eating measures. Others found, a larger positive effect in high-risk participants, over the age of 15 years, and female-only participation groups [3], [4]. However, usually effect sizes from effectiveness trials were small to moderate, though some effects were medium in magnitude [1].

It was suggested that school-based programs should include strongly interactive and participatory components [2], [5] and incorporate self-esteem components [6]. To avoid glorification of eating disorder, a wellness approach may be preferred [7]. A wellness approach to prevention programs focuses on overall aspects of well-being, addressing self-esteem, problem solving, stress management, and goal setting, with no special attention to eating disorders.

In Favor of Myself addresses the four main areas that were found effective when incorporated in preventive programs [7]: Education about adolescence, media literacy [8], [9], cognitive dissonance [1], [3], [10] and incorporation of wellness approach [5]–[7]. The overall impact of “in Favor of myself” and the study protocol were published previously [10].

A frequently asked question is whether an intervention should incorporate boys and girls together or should be gender specific. This question has not been addressed specifically so far in the research field.

Physical, neurological and psychological changes are often experienced differently by male and female adolescents. It has been observed that men assess their physique in terms of strength (chest, shoulders, biceps and muscular strength), while women express concerns related to weight and the shape of certain body parts (hips, thighs, buttocks, etc.).

Concern for body image differs according to the gender. While boys are concerned with having a muscular body image, girls have beauty ideals that are inseparable from thinness, in most cases below a healthy size [8], [12]. So, for men, being underweight is seen negatively but for women it is desired [13].

Interestingly, some research found that for men, thinking you are less muscular than you really are, is related to higher depression scores and eating disorder symptomology [14].

Amongst the sociocultural factors that influence most negatively female and, progressively more, male body image perceptions, are stereotypical ideal body representations, transmitted through different socialization agents and most notably the mass media [15].

Although girls have more correct BMI than boys, girls show greater prevalence when it comes to suffering misperceptions of their body image and this creates a greater number of behaviors dedicated to control their weight [16]. For example, the percentage of adolescents who skip breakfast is higher among girls than among boys; that percentage, in many studies, is more than double [17], [18].

Although females, more likely than males, might experience eating disorder symptoms and associated risk factors like thin-ideal internalization and self-objectification, there are also many men who feel dissatisfied and concerned about their physical appearance, and may be affected by eating disorders or use steroids [19]. Therefore, gender discussions should be included within studies evaluating the effectiveness of prevention programs.

The majority of prevention interventions had a focus on the needs of females [20]. However, research does indicate that boys do have body image concerns which may result in body dissatisfaction, steroid use and body dysmorphic disorder [1, 19)] It has been recognized that it is important that attitudinal change takes place in boys as well as girls to support change [2]. Thus, a number of studies did include boys in the interventions [6], [21]–[23]. O’Dea and Abraham [6] reported that the ‘Everybody’s Different’ program was significantly beneficial to male as well as female students. An average of 87% of male students reported that the education program had been of value to them and 72% indicated that they would like to be involved in another similar education program if it becomes available in the future. However, when analyzed separately, the program had no impact on body satisfaction or drive for thinness in males. González et al [23] reported that a media literacy based program revealed, at the long term follow-up (thirty months), an effective internalization of the aesthetic body ideal on boys and girls as a way of sustaining resilience. The authors also found improvement among girls, as well as boys, that at baseline scored the “EAT-26” above the cut-off [23]. This manuscript describes the gender differences in respect to self-esteem, self-image, body-image, media literacy and contingencies of self-worth among adolescents who participated in a school-based wellness program, In ‘Favor of Myself’. This program focuses on self-esteem, self- image, body-image, media literacy and cognitive dissonance, due to their perception as protecting agents against hazardous behaviors such as eating disorders, alcohol abuse and drugs [8].

Our study explored the genders' differences responses to ‘In Favor of Myself’, a wellness program that was evaluated via a longitudinal, controlled intervention study.

## Methods

The study was approved by Tel Hai institutional research board and every parent signed a written consent.

### Participants

Two hundred and ten adolescents (mean age 13.5, ranged between 12–14 years) participated in the intervention group. All adolescents in the studied school participated in this study. Only those who filled out the questionnaire on at least two occasions, were analyzed for the evaluation of the program. No differences were observed in baseline variables between those who filled the questionnaire on two or three occasions and those that did not (filled only on baseline) [10].

For the purpose of discussing the gender differences in respect to the program impact, only the intervention-group data will be reported according to gender. The overall results of the intervention vs. control were previously published [10].

### Program and process description

Detailed description of program and process is given in previous publication [10]. In short, ‘In Favor of Myself' consists of eight structured 90-minute sessions, delivered approximately one week apart in groups of 15–20 participants. Each session describes the background of the topic and offers interactive activities to engage participants in the subject in both verbal and nonverbal ways. The program was integrated into a regular school coping skills curriculum- a program which offers teachers with a list of topics and activities to deliver in each grade. It is delivered once a week, focusing on personal and social skills.

After receiving Tel Hai Institutional review board approval and parents' informed consent, the baseline status of participants and controls were assessed using a computerized questionnaire which was filled also at program termination and at 3 months follow-up. Twenty trainers (school counselors and teachers) were trained to deliver the program while the task of data collection was assigned to research students. Each participant was given an identification number to ensure confidentiality and anonymity.

### Measures

Demographics, including personal and familial details, were obtained from each participant at baseline. The psychometric properties of the self-report measures were previously described [10]. In short, the computerized questionnaire included the following measures: perception of the changes occurring during adolescence, identification of media strategies that promote consumption and internalization of stereotypes, pressure by media using the pressure subscale of SATAQ-3, contingencies of self-worth using the other's approval and appearance subscales of CSW), self-esteem using the Rosenberg self-esteem scale, body image using the figure body images (FBG), eating attitude and behaviors using EAT-26 and drive for thinness and body dissatisfaction which are subscales from Eating Disorders Inventory-2.

### Data analysis

Participants were clustered in 9 program-groups. In order to take into consideration the unit treatment additivity the intraclass correlation coefficient (ICC) was calculated for each variable. Due to reasonable ICC (range of 0.03–0.07) the analyses were performed with all data sets together.

The change in scores over time was analyzed after the intervention (pre->post) and over the 6 months study (pre->3 months follow-up). One way analysis of variance (ANOVA) and χ^2^ tests were utilized to assess comparability between boys and girls on baseline measures.

2×3 MANOVA (gender × time) with repeated measure on time was used to assess the difference between genders in changes over time. Statistical analysis was conducted only for those who completed the questionnaire in the three assessment times (210 out of 300 students). No differences in baseline variables were observed between those who filled the questionnaire on three occasions and those that did not (filled only on two occasions).

Effect size is described using partial **η^2^** (Partial eta-squared) where 0.01 constitutes a small effect, 0.059 a medium effect and 0.138 a large effect [24]. The statistical analyses were undertaken using SPSS computer program (SPSS, Chicago, IL) for Windows and a p<0.05 was considered to be statistically significant.

## Results

### Participant characteristics

For the purpose of discussing gender differences and since the intervention vs. control conditions differences were presented in previous manuscript, we will present and discuss hereby the gender differences among the intervention group participants only.

The intervention group included 210 participants, 55% girls and 45% boys, aged 12-14 years old (Table 1).

**Table 1:** Participants demographic data at baseline according to genders.

Variable	Intervention (n = 210)		
	BOYS N = 96	GIRLS N = 114	p/χ^2^ between genders
**Gender** (%)	45%	55%	
**Age** (mean years± SD)	0.86±13.5	13.12±0.97	NS
**Country of birth** (number, %)			NS
Israel	89 (96.7%)	106 (90.0%)	
Other	3 (3.3%)	12 (10.2%)	
**Family type (%)**			NS
Parents married	92%	83.5%	
Other (divorced or one parent)	8%	16.5%	
**Number of siblings (%)**			NS
≤ 4	81%	97%	
>5	19%	3%	
**Father's education level (%)**			NS
Academic degree	31%	36%	
Vocational education	28%	22%	
High school	41%	42%	
**Mother's education level (%)**			NS
Academic degree	51%	54.2%	
Vocational education	17.5%	20.8%	
High school	31.5%	25.0%	
**Economic status (%)**			NS
High	35.7%	39.%	
Medium	64.3%	56.4%	
Low	0%	4.3%	

(Mean ± SD, N and % of population).

No statistically significant differences were found between genders in the intervention group in respect to mean age, country of birth, family size and socioeconomic status (assessed by parents' occupation and education).

The differences between boys and girls at baseline and at program conclusion in respect to awareness to changes associated with getting older, Rosenberg self-esteem, Others' approval and appearance contingencies of self-worth, knowledge about advertising strategies as well as impact of media on pressure to change oneself and current vs. ideal body image will be described.

### Awareness to changes associated with adolescent and getting older

At baseline, there were statistically significant differences between genders in awareness to adolescents changes [MANOVA: F(2,202) = 4.8, p = 0.003, partial η^2^ = 0.068). Girls reported on being more aware to life burdens and at the same time perceiving life as more exciting and interesting.

At program conclusion, MANOVA revealed a significant change with large size effect in both genders' perception about adolescence [F(2,202) = 35.8, p = 0.000, partial η^2^ = 0.38]. Gender × time effect was also statistically significant (F(2,202) = 3.23, p = 0.02, partial η2 = 0.053) mainly due to girls reporting on higher increase in perception of life as exciting and interesting when getting older.

At follow-up, MANOVA revealed a significant change with large size effect size in both genders perception about adolescence [F(4,202) = 14.99, p = 0.000, partial η^2^ = 0.45]. Gender × time did not have a significant effect.

### Self-esteem, contingencies of self-worth and self-image

#### Rosenberg self-esteem

Baseline analysis revealed statistically significant differences in self-esteem between genders [F(1,210) = 12.5, p = 0.001, partial η^2^ = 0.058). Girls reported on 20% higher in Rosenberg self-esteem scale compared to boys. No difference has been noted in self-esteem among participants due to the intervention, nor due to time × gender.

#### Contingencies

**Figure 1:**
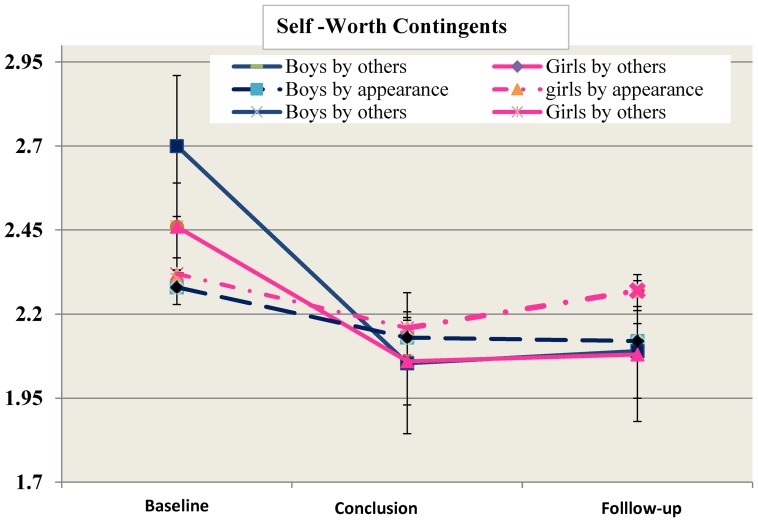
Contingencies of self-worth by others and by appearance (Means ± SD).

Baseline analysis also revealed significant differences in contingencies of self-worth by appearance between genders (F(1,210) = 13.03, p<0.000, η^2^ = 0.061) with girls attributing 15% higher importance to contingencies of self-worth by appearance compared to boys. At program conclusion, as well as at program follow-up, a significant reduction (from baseline) has been noted in contingencies of self-worth by appearance in both genders (F(2,208) = 3.99, p = 0.02, η^2^ = 0.033] with no time × gender impact (Figure 1).

In respect to contingencies of self-worth by others, at baseline, analysis revealed no difference between genders. At program conclusion a significant reduction has been noted in both genders with large effect size (F(2,210) = 40.6, p = 0.000, η^2^ = 0.25]. Moreover, a significant gender × time effect was found in program impact between baseline and program conclusion [F(1,210) =  5.3, p = 0.02, η^2^ = 0.03] Boys exhibited greater reduction in contingencies of self-worth by others, than girls. No gender × time impact has been noted between baseline and follow-up. (Figure 1).

Gender differences were also noted when adolescents were asked to estimate on a scale of 1 to 4, the influence of different factors on their self-image (Table 2).

**Table 2:** Means, Standard deviations and Univariate analysis of factors influencing self-image.

Contribution to self image of:	Baseline - (T_1_)			Conclusion - After 3 months (T_2_-T_1_)			3 months follow-up - (T_3-_T_1_)		
	Boys N = 96	Girls N = 114	p_1_	Boys N = 90	Girls N = 110	p_2_, η^2^ GenderXTime	Boys N = 98	Girls N = 114	p_2_, η^2^ GenderXTime
Parents' perception	2.75±1.1	2.60±0.9	NS	2.51±1.0	2.83±0.9	NS	2.67±1.0	2.87±1.0	0.02, 0.05
School achievements	2.46±0.9	2.36±0.9	NS	2.3±0.9	2.48±0.9	NS	2.46±0.9	2.58±1.0	0.01, 0.056
Appearance	2.37±0.9	2.91±0.9	0.000	2.2±0.9	2.62±1.0	NS	2.21±1.0	2.43±1.2	0.01, 0.06
Popularity	2.25±0.8	2.45±0.9	0.02	2.20±0.8	2.4±1.0	NS	2.1±0.8	2.4±0.9	NS
Admired people	1.60±0.8	1.88±0.9	0.03	1.60±0.8	1.74±0.8	NS	1.60±0.9	1.7±0.7	NS

p_1_ =  significance of differences between genders at baseline.

p_2_  =  significance of Time × Gender impact.

At baseline, girls reported on statistically significant higher values, compared to boys, in their perception of the level of the impact of popularity (14%), appearance (27%), and impact of admired ‘heroes’ on their self-image (18%) (Table 2). Boys perceived parents' perception as the primary contributor to their self-image while girls considered appearance to be the primary contributor. Both genders considered admired people to be the lowest contributor.

At program follow-up statistically significant effect of time × gender has been found in respect to the importance attributed to the impact of achievements at schools [F(1,210) = 5.77, p = 0.01, partial η^2^ = 0.056], parents perceptions [F(1,210) = 5.07, p = 0.02, partial η^2^ = 0.05] and appearance [F(1,210) = 6.2, p = 0.01, partial η^2^ = 0.06] (Table 2). Following the program, girls reported on reduction in the attribution of appearance to their self-image and increase in contribution of school achievements and parents' perception. Boys did not show this trend. Significant gender × time effect with medium size effect has been noted in respect to the attribution related to appearance (Table 2).

### Media literacy knowledge and impact

**Figure 2:**
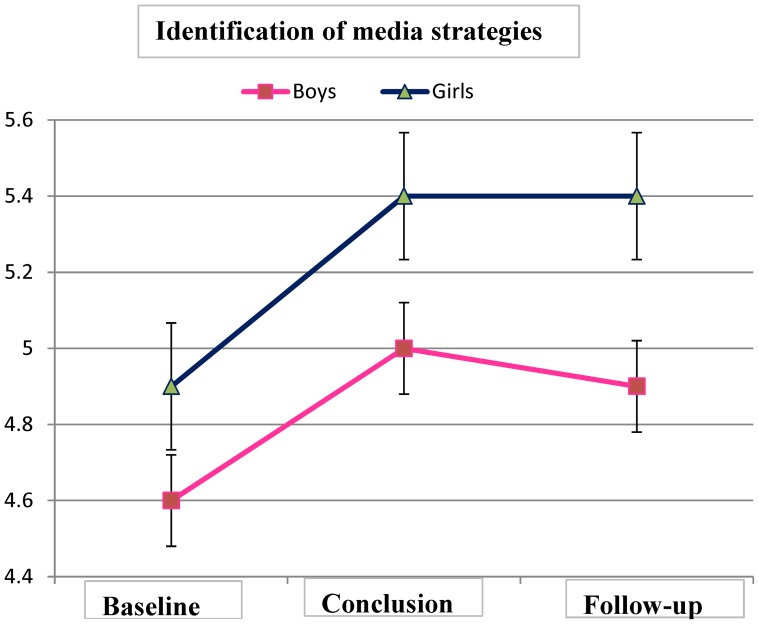
Identification of media strategies by girls and boys (Means ± SD).

In respect to identification of media strategies that are endorsed with advertisements, no differences were found between genders at baseline and at program conclusion. A statistically significant time effect with large effect size had been found between program conclusion and baseline in both genders (F(1,206) = 26.6, p =  0.000, partial η2 = 0.12) but no gender × time effect. The improvement in identification of media strategies remained at follow-up (Figure 2).

In respect to awareness to the pressure to “fix” oneself, endorsed by the media, at baseline, significant differences were found between girls and boys [F(1,206) = 22.8, p<0.000, partial η^2^ = 0.10). Girls reported to be more influenced than boys by the media.

At program conclusion analysis revealed, in both genders, a significant improvement (reduction in induced pressure) with medium effect size [F (2,206)  = 6.15, p = 0.01, partial η^2^ = 0.049].

However, statistically significant differences were not found in the way the program impacted boys vs. girls in this variable.

### Body image and attitudes

#### Body image

Body image was measured using The Figure Body Images (FBG) [25].

Participants were asked to score silhouettes in respect to each of the 6 variables: current body image, ideal body image, attractive body image of both genders and healthy figures of both genders. Mean ratings and standard deviations of participants' body figures according to gender are presented in table 3.

**Table 3:** Mean and standard deviations of body image figures chosen by girls and boys.

	At baseline - Mean ± SD			AT termination - Mean ± SD			At 3 months follow-up - Mean ± SD		Intervention impact (time) F,p,partial η^2^	Gender × Time F, p partial η^2^
Figure	Boys	Girls	*P	Boys	Girls	*P	Boys	Girls	All Group	
Current figure	3.93 ±1.3	3.80 ±1.1	NS	3.93±1.3	3.70±0.9	0.00	3.65±1.3	3.70±0.9	NS	NS
Ideal figure	3.88 ±1.4	2.93 ±1.0	0.00	3.88±1.3	2.98±0.9	0.00	3.47±0.9	3.11±0.7	3.36,0.03;0.037	4.95,0.02;0.054
**Gap = current-ideal**	−0.05	+0.87	0.00	−0.05	+0.72	0.00	−0.18	+0.59	NS	NS
Girl's attractive figure	4.05±2.0	*3.15 ±1.3*	0.00	3.65±2.0	3 *3.13±1.2*	0.00	4.05±1.9	*3.24±1.1*	NS	NS
Boy's attractive figure	*4.16±1.8*	3.37±1.3	0.00	4 *3.79±1.9*	3.28±1.2	0.00	*3.98±1.8*	3.46±1.1	NS	NS
**Gap = current-attractive**	−0.12	+0.65	0.00	*−0.14*	+0.57	0.00	*−* 0.33	+0.46	NS	NS
Girl's healthy figure	4.35 ±2.2	*3.43 ±0.8*	0.00	4.19 ±1.7	*3.70 ±0.7*	0.00	3.88±1.5	3.57±1.1	NS	NS
Boy's healthy figure	*4.53 ±2.3*	3.50 ±0.9	0.00	*4.16 ±1.9*	3.72 ±0.8	0.00	4.02±1.7	3.63±1.1	NS	NS
**Gap healthy-current**	*−* 0.60	+0.37	0.00	*−* 0.23	0.00	0.00	*−* 0.37	+0.13	NS	NS

*P -Significance of difference between genders.

MANOVA revealed a statistically significant and large effect size of gender on body image perceptions (F(6,205) = 4.69, p = 0.000, partial η^2^  = 0.25).

**Figure 3:**
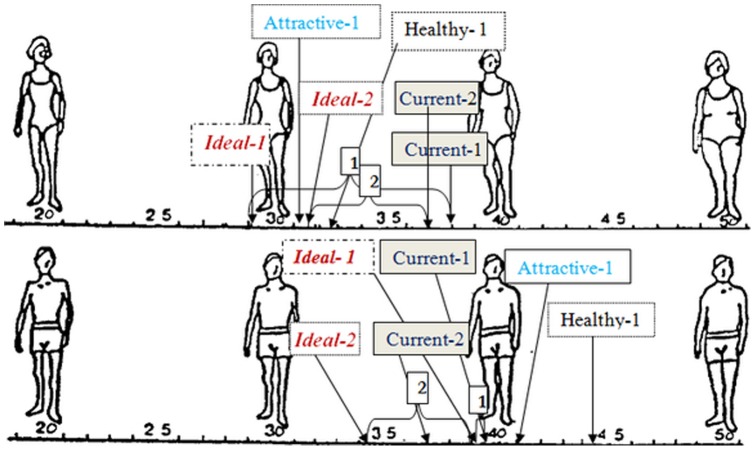
Mean and standard deviations of body figures rating of adolescents: girls (top) boys (bottom).

At baseline there were statistically significant differences between genders in perceptions of ideal self-figure, attractive figures and healthy figures (Table 3). Boys perceived their current body figure almost the same as their ideal, attractive and healthy figures while girls perceived their current body figure as substantially higher than their ideal, attractive and healthy figures. The relative placement of the current body figure of girls (top) and boys (bottom), in respect to the ideal, attractive and healthy figures is presented in Figure 3. Time had significant effect only on perception of ideal body figure (Table 3) [Univariate: F(2,205) = 3.36, p = 0.03; Partial η^2^  =  0.037).

At baseline, the mean ideal body figure of girls in the intervention group was 2.93±0.8, at program conclusion it increased to 2.98±0.7 and at the 3 month follow-up it further increased to 3.11±0.7. The difference between T_3_ and T_1_ in respect to perceived ideal body figure was statistically significant (Univariate: F(2,205) = 4.95, p = 0.029; Partial η^2^  =  0.054).

In respect to the gap between current figure and the ideal, attractive and healthy figures, there was a consistent difference between the genders. Girls always perceived their current body figure *above* their ideal figure as well as *above* girls' attractive and even healthy figures, while boys always felt their current body figure is *below* their ideal as well as *below* boy's attractive and healthy figures (Figure 3).

This trend was observed during all three assessment points. The impact of the intervention was different among genders, but it did not reach a statistically significant effect.

In contrast to boys who had demonstrated, during time of assessments, increase in the gap between current figures vs. ideal or attractive figures, in girls the gap decreased.

Nevertheless, in both genders the gap between perceptions of current figure and healthy figure decreased. In girls it even reached 0 at program termination.

#### Eating disorders features

At baseline, MANOVA revealed significant impact of gender on body dissatisfaction and drive for thinness [F(2,204) = 6.9, p = 0.001, partial η^2^  = 0.066). Girls reported on statistically significant higher scores than boys in both EDI-2 scales: body dissatisfaction [F(1,204) = 14.5 p = 0.002, partial η^2^  = 0.047) drive for thinness scale [F(1,204) = 22.9, p = 0.000, partial η^2^  = 0.066).

At program conclusion MANOVA revealed a statistically significant reduction in EDI in both genders with medium effect size [F(2,204) = 6.23, p = 0.002, partial η^2^  = 0.07] as well as significant gender × time effect [F(2,204) = 3.23, p = 0.04, partial η^2^  = 0.04]. Univariate analysis revealed a medium size effect of time on drive for thinness [F(1,204) = 4.64, p = 0.006, partial η^2^  =  0.044] and no effect in body satisfaction [F(1,204) = 4.64, p = 0.46, partial η^2^  = 0.003] as well as low gender × time effect [F(1,204) = 2.48, p = 0.04, partial η^2^  = 0.02].

At program follow-up, MANOVA revealed a statistically significant reduction in EDI in both genders with large effect size [F(4,204) = 3.5, p = 0.01, partial η^2^  = 0.12). No gender × time effect had been noted.

## Discussion

The current study aimed to explore the gender differences in respect to the impact of “In Favor of Myself” - a widely disseminated wellness program to promote positive attitude towards growing, positive sense of self and self-esteem and positive body image among adolescents. The program also aimed to promote ability to filter external media and culturally inappropriate messages about diversity of beauty.

Overall, the results of this study support and extend previous research suggesting that there are gender differences in respect to body image and self-image in young adolescents. Further, there appear to be some differences but some similarities in how girls and boys are affected by preventive program.

Gender differences were statistically significant, already at baseline, in most of the variables. Compared to boys, girls reported to be more contingent by appearance, and that their self-image is more influenced by popularity, appearance, interpersonal communication and admired people. These findings seem to agree with the results of most studies conducted on gender and its relation with appearance, achievement and with self- efficacy and self- esteem [26]. Girls reported on more awareness to life burdens and at the same time perceivedlife as exciting and interesting at baseline.

In respect to body image, a larger gap has been noted among girls between current body figure and perceived ideal figure at baseline as well as higher awareness to the pressure imposed by media and higher wish to ‘fix’ oneself. Not only were they more dissatisfied with their body, but girls were more active in attempts to become and/or remain “thin”.

Researchers support the idea that there are gender disparities in adolescent self-esteem with some claiming that girls having lower self-esteem [27]–[29] and others found that boys have lower self-esteem [30]. These differences might be explained by the different ages and cultures investigated. The higher self-esteem in boys compared to girls is more common in middle adolescence than in early adolescence, as was found in our study.

Studies reported by Fairburn indicate that when adolescent girls are insecure, they become significantly more self-conscious and have greater concerns about popularity, lower body esteem, and lower self-esteem. Self-consciousness leads to increased self-criticism, leaving the adolescent extremely vulnerable to disordered eating [31]. Another explanation is that girls are more liable than boys to be influenced by their physical self-esteem [32], which has been found to be central to overall self-esteem [27]. Oliva [33] assumes that these differential patterns of self-appraisal have their origins partly in parental gender linked beliefs and partly in cultural stereotypes.

Young girls are searching for positive themes with which to identify, to make them feel like they fit in with a group and to make them feel confident. Ideally, the teenager will emerge from the process stronger and more confident, knowing who they are and thinking they know what they want out of life. Unfortunately, this is not always the case [28].

The large effect size of gender at baseline questions the appropriateness of providing universal preventive programs to mix gender groups.

At program termination, gender × time effect was detected with only girls exhibiting significant increase in awareness to changes associated with adolescence, reduction in contingencies of self-worth by others, increase in importance given to achievements at schools, parents' perceptions and decrease in importance given to appearance in relation to self-image. Boys did not show this trend. A large effect size (partial η^2^ = 0.38) with statistically significance had been found in awareness to changes associated with adolescent due to the intervention but with only small gender × time effect (η^2^ = 0.053). Girls reported on larger increase in perception of life as exciting and interesting when getting older. Most differences between girls and boys were also detected at follow-up.

These changes demonstrate a process of ‘growing’, acquiring knowledge and change in perceptions following the intervention. Although knowledge may not immediately translate into behavioral changes, it may well provide protection against external pressures at a later date, and provide a foundation on which to evaluate new situations.

In respect to body image, dissatisfaction and drive for thinness, a statistically significant increase has been noted in the size of ideal body figure among girls in the intervention group as well as decrease in the gap between current and ideal body figure. The change in ideal body figure, was gender sensitive, with higher and maintained change in girls (assessed at 3 months follow-up), while smaller and disperse among boys. Both genders exhibited reduction in drive for thinness with no gender × time effect.

Other studies employing the Figure Body Images also showed that in the case of girls the ideal body is substantially smaller than their own, whereas no difference in choice occurred in the case of boys [34]–[36]. Owing to a more critical stance by girls with regard to their body image, they manifest a high esteem for their body image which causes a self-awareness related to overweight and obesity. Nevertheless, the presented intervention succeeded in decreasing the gap between current and ideal body figure of girls in the intervention group. In contrast, boys mean current figure at baseline was closer to their perceived ideal figure and lower than their perceived healthy figure. In respect to the gap between current figure and the ideal, attractive and healthy figures, there was a consistent difference between the genders. Girls always perceived their current body figure above their ideal figure as well as above girls' attractive and even healthy figures, while boys always felt their current body figure is below their ideal as well as *below* boy's attractive and healthy figures. This trend had been observed during all three assessment point. The impact of intervention on body image was different among genders, but it did not reach a statistically significant effect. In contrast to boys who had demonstrated, during time of assessments, increase in the gap between their current figure vs. ideal or attractive figuresm, in girls the gap decreased. Since the change in boys' gap was not statistically significant it is hard to draw conclusions in respect to these gender opposite trends. Although research documents that some adolescent boys experience body dissatisfaction, these concerns appear to be less pronounced for boys than they are for girls in this age range and less than those of adult males. Hargreaves and Tiggeman [37] found that among adolescent boys aged 14 to 16, a small percentage of boys reported high levels of body image dissatisfaction and engagement in body change strategies. Most of the boys discussed a desire to change their weight and their height, though the most prevalent body image concern was about being stronger and more muscular. O’Dea & Abraham [6] reported that post-pubertal boys have been shown to have less body dissatisfaction then prepubertal boys.

Moreover, the use of body figure ratings to assess men's body image dissatisfaction is problematic because many men want to become more muscular rather than fatter but the figure ratings do not allow them to express this goal. The use of these figure rating scales could explain why previous research has found that men and boys experience fewer body image problems. Most males are not likely to indicate a desire to look like a chubbier figure than they already are (except males with a low BMI) when their real desire is to become more muscular.

Flament et al [38] reported that in adolescent males, weight-esteem partially mediated the relationship between muscular ideal and restrained eating; while appearance-esteem partially mediated effects in the emotional and external eating regressions. This finding suggests that boys may be more at risk to engage in restrained eating behaviors when their focus is on their weight, and not their overall appearance, in relation to the lean, toned look portrayed in the media. Moreover, Flament et al [38] suggested that it could be that poor appearance-esteem and internalization of the muscular ideal are more likely to occur among males who experience negative affect and emotional instability. In both genders, appearance-esteem appears as the sole (males) or stronger (females) predictor for both emotional eating and external eating. Appearance-esteem includes qualities beyond weight satisfaction, e.g., satisfaction with muscle tone and facial beauty. Perhaps the additional qualities of appearance-esteem account for the dominant association with emotional and external eating, which offers an opportunity for future research in the field.

As has been previously reported by us [11] reduction in influence of appearance on self-worth was found to be associated with self-esteem. Those with higher self-esteem at baseline showed greater reduction in influence of appearance on self-worth. Brown [39] suggested that global self-esteem guides the way people evaluate their specific qualities. From this perspective, people who are fond of themselves in a general way (i.e., those with high self-esteem), imbue themselves with many positive qualities. They like the way they look and they appreciate more their talents [40].

Thus, preventive program should incorporate contents that address global self-esteem as well as appearance-esteem and not only focus on weight-esteem.

Since our study has showed that boys exhibited fewer gains from the program in comparison to girls and showed a tendency towards increase in the gap between current figure and ideal or healthy figure, it is very important to explore whether prevention intervention programs may actually have a counter-productive effect on boys and may be girls might gain more from the program when the program is delivered in mixed gender groups. Since boys tend to be less emotionally matured than girls, and less self-conscious about their appearance in mid adolescents years, (when this program is delivered), a long-term follow up should be performed to observe the program impact for both genders.

Paxton [2] suggested that it may well be that girls, perhaps more particularly older girls, will feel inhibited and vulnerable discussing body image issues in the company of boys, and this would have a negative impact on those who arguably need the program most. In addition, the issues for boys, e.g. bulking up and gaining height, may not be very relevant for girls and better be managed in programs specifically tuned for boys. Furthermore, different maturational levels of boys and girls may present difficulties for mixed discussions on some issues. On the other hand, boys contribute in a very powerful way to the social environments of girls and vice versa, so awareness of each group on the pressures on their counterpart may well be beneficial. Gonzalez et al [23] reported that in their program both boys and girls exhibited improvement in internalisation of the aesthetic body ideal, thirty months after termination.

Limitations of the present study must be acknowledged. Self-report measures may fail to provide reliable information, mainly in respect to eating behaviors and to body misperceptions since we did not use anthropometrical parameters. Moreover, due to limited resources, longer-term follow-up with the current population was not possible. Despite such limitations, the study's longitudinal nature, the intervention that targets multiple risk factors (media images, stereotypes, interpersonal communication, peer influence, media literacy and critical perspective towards the ideal self and body image) and the gender × time analysis are major advantages which may provide causal associations among the variables examined.

Moreover, it provides information on both male and female adolescents, which enable us to focus on the gender differences and consider which topics might be extended for each gender.

Given the differences in individualization-separation processes and ideal body image for males and females, males report pressure for increased masculinity and higher BMI [22], it is essential to investigate principles for developing healthy body satisfaction for both genders. Further research is needed to explore the long term impact of this program as well as its impact when provided to girls and boys separately.

In conclusion, the main objectives of prevention programs conducted in schools should be to identify and criticize the aesthetic beauty model, to develop critical thinking skills and to challenge the glorification of thinness for girls and muscular ideal for boys [8] as well as support self- esteem which relies on other foundations than appearance.

Since changes brought about by interventions are typically, though not always, short-lived, it is insufficient to have one intervention and then no further support of the ideas and attitudes introduced in the program. Most likely, developmentally appropriate interventions should continue to be introduced throughout childhood, adolescence and young adulthood. Continued interventions or booster sessions need to be considered mainly in light of the reports that programs that were offered to participants over 15 years of age had a greater effect [3] than those offered to younger ones.

Today, there is no reliable evidence supporting the superiority of either mixed or uni-gender groups thus future studies may benefit from comparing the impact of the program when delivered to mixed gender groups vs. uni-gender groups as well as longer-term follow-up.
